# Understanding Health Information Seeking from an Actor-Centric Perspective

**DOI:** 10.3390/ijerph120708103

**Published:** 2015-07-15

**Authors:** Simon Batchelor, Linda Waldman, Gerry Bloom, Sabrina Rasheed, Nigel Scott, Tanvir Ahmed, Nazib Uz Zaman Khan, Tamanna Sharmin

**Affiliations:** 1 Gamos Ltd, Reading, UK; E-Mail: research@gamos.org; 2 Institute of Development Studies, Brighton, UK; E-Mails: L.Waldman@ids.ac.uk (L.W.); G.Bloom@ids.ac.uk (G.B.); 3 International Centre for Diarrhoeal Disease Research, Bangladesh; E-Mails: sabrina1@icddrb.org (S.R.); tanvir@icddrb.org (T.A.); nazib@icddrb.org (N.U.Z.K.); tamanna@icddrb.org (T.S.)

**Keywords:** health information seeking, ICT, mobiles, Bangladesh

## Abstract

This paper presents a conceptual approach for discussing health information seeking among poor households in Africa and Asia. This approach is part of a larger research endeavor aimed at understanding how health systems are adapting; with possibilities and constraints emerging. These health systems can be found in a context of the changing relationships between states, markets and civil society in low and middle income countries. The paper starts from an understanding of the health sector as a “health knowledge economy”, organized to provide people with access to knowledge and advice. The use of the term “health knowledge economy” draws attention to the ways the health sector is part of a broader knowledge economy changing the way individuals and households obtain and use specialist information. The paper integrates an actor centric approach with the theory of planned behavior. It seeks to identify the actors engaged in the health knowledge economy as a precursor to longer term studies on the uptake of innovations integrating health services with mobile phones, commonly designated as mHealth, contributing to an understanding of the potential vulnerabilities of poor people, and highlighting possible dangers if providers of health information and advice are strongly influenced by interest groups.

## 1. Introduction

This paper examines health information seeking in Bangladesh from an actor-centric perspective. This is part of a larger research endeavor aimed at understanding the pluralistic health systems, which have emerged in a context of changing relationships between states, markets, and civil society in many low and middle-income countries [1]. While many innovations integrating health services with mobile phones, commonly designated as mHealth, build on innovations in information and communication technologies (ICT), and seek to use them as mechanisms for delivering health advice and health information, few initiatives start with user demand. This paper maps the key actors within this health knowledge economy, and explores how ICT could be used to enhance or adjust health information seeking behavior. It then asks how we can strategically strengthen or adapt such behaviors and how we can use knowledge of such behaviors to develop ICT-related health interventions to help the poor and vulnerable.

In the actor-centric approach outlined below, we model the complexity of the health system, as well as show the intricacies associated with information seeking and decisions about treatment. Health provision is not a linear flow from government to citizen. Rather, as Bloom and others have stated [2], the health system in emerging economies, is a mix of government and private provision, formal and informal providers, physical and informational provision, and informed and uninformed commentary. Health provision in Bangladesh is embedded within a health knowledge economy, in which access to information is shaped by power differentials, and in which informal relationships are extensively drawn upon to help overcome these differentials.

In this paper, we analyses aspects of the health sector as a knowledge economy, organized to make the benefits of widely available medical technology, such as drugs or medical advice [2]. This applies particularly to health services that rely on the provision of advice and treatment on an outpatient basis. We hypothesize that the spread of ICTs could lead to substantial changes in the ways people seek health information and care [3,4], with the potential of a significant impact on poor people, whose wellbeing is strongly influenced by the degree to which they have access to safe, effective and affordable health services [5].

The view of the health sector as a “health knowledge economy” differs from the more common understandings of it as an assemblage of human resources and goods and services. It draws attention to the ways that knowledge is imparted, to differential access to knowledge, and to the power relations that govern interactions in which knowledge is distributed [2]. It also situates the health sector within a broader knowledge economy, which is changing the way individuals and households obtain and use specialist information. Within knowledge economies, a defining feature is the creation and use of knowledge as a mechanism for wealth generation [6]. A major influence on the organization of health systems is the asymmetry of information between the providers and users of expert medical knowledge [7]. This has resulted historically in the creation of self-regulating professions, a variety of accreditation mechanisms and government provision of services to ensure that experts are competent and act in the interests of their clients. These arrangements are under pressure because of a growing tendency for people to bypass specialist “gatekeepers” to knowledge, such as trained professionals, and seek information, advice and goods, such as drugs, either directly or via non-regulated providers.

In this context, new ICTs are disruptive technologies, with the potential to lead to substantial changes in the ways people seek health information and care [4,8]. Recent studies have pointed to the relevance of new ICTs to economic development [9,10] and poverty alleviation [11,12,13]. The wellbeing of poor people is strongly influenced by the degree to which they have access to these new sources of information [14].

The paper begins with an overview of the changes underway in health knowledge economies, focusing particularly on mobile phones and their use for mHealth services. mHealth is a label used to refer to the ways in which technological and portable devices, such as mobile phones, personal digital assistants and others are used in health [15] as a means to support community health workers and enhance data collection [16]. eHealth concerns the use of ICTs for the improvement of health care and health service delivery. The World Health Organization (WHO) [17] describes eHealth as “the transfer or health resources and health care by electronic means” and as encompassing the sending of health information messages (to health professionals, health workers and patients); the use of ICT to enhance public health services and the utilization of electronic devices, developed for commerce and business, in health systems management [17].

Drawing on Batchelor *et al.* [18], the paper then presents an actor-centric approach for discussing health information seeking behavior by the poor, using an example from qualitative research to illustrate its application. The paper integrates this approach with the theory of planned behavior, which leads one to pose critical questions such as: Who holds the trust or respect of the people enough that they might act upon advice? What channels of information are used by the public? Where are people seeking their health information, both passively and actively? Addressing these questions helps illuminate new ways in which ICTs might be used to support health systems and for developing new applications which seek specifically to meet the needs of the poor and disenfranchised.

### Methods

The paper draws on findings of an ongoing study of health information seeking by households in Bangladesh. The study is a mixed methods exploration of changing patterns of health information seeking behavior (at individual and household levels) in the context of a rapidly changing knowledge economy. The study has undertaken literature reviews of health information seeking, telemedicine businesses and of ICTs and health, as well as reviews of policy processes. Stakeholder interviews were conducted with key private and public actors. A research team undertook field studies using both qualitative (35 interviews and 6 focus groups) and quantitative methods, surveying 2556 households using cluster randomized sampling within the 3 geographic locations; Chakaria (a relatively remote district); Mirzapur (a rural setting with good road access to Dhaka); and Korail slum (in Dhaka city). Insights developed through the qualitative interviews and focus groups fed into the development of the quantitative survey. The survey questions asked extensively about both hypothesized and actual behavior in relation to illness and health information seeking. Analysis included examining predictor variable, *i.e.*, descriptors of resource allocation, ownership and access to ICT, beliefs about the health of the family, access and involvement with social networks, availability of social capital, awareness of health information on the media, and the degrees of trust and value placed on each source. The Ethical Review Board of the International Centre for Diarrhoeal Disease Research, Bangladesh (hereafter icddr, b) provided ethical clearance for the research PR:13026.

## 2. The Changing Health Knowledge Economy

The main premise of the study is that change is afoot in the health knowledge economy in Bangladesh. This is partly being driven by advances in ICTs and rapid developments in mobile phone markets [19,20]. These changes have implications for actors primarily engaged in the provision of health services and also for mobile network operators (MNOs), and the regulatory bodies charged with protecting the interests of the poor.

### 2.1. Changes in the Complex Health Knowledge Economy

Bangladesh has a pluralistic health system with a wide variety of service providers in terms of the knowledge system they adhere to, their level of training and their relationship to the public sector and the regulatory system [21]. For those who can afford it, there is a sophisticated market with private hospital facilities and professional services. The public sector provides basic health services to the rural population at upazila health complexes, based at Bangladesh’s second lowest tier of regional administration. These are relatively small facilities with around 30 in-patient beds and basic laboratory facilities, staffed by a small number of medical doctors, and supporting staff [21]. These complexes are supplemented by sub-centers at Union level, the lowest administrative unit. The sub-centers are staffed by medical assistants and midwives who provide health care to outpatients in the form of consultations, treatments or other interventions. These centers provide Bangladesh’s Essential Health Care package, which incorporates maternal health, family planning, communicable disease control, child health, and basic curative care [22]. A network of community health workers (Shasthya Sebikas), some of whom are employed by NGOs, some of whom are voluntary and some of whom hold government posts, are trained to support the upazila health complexes through home visits which promote and provide health and family planning services.

Considerable barriers in this formal-yet rather small or limited–health system reinforce the high reliance on informal and private health services [23]. A very high proportion of health-related consultations are with informal providers and drug sellers rather than with the formal health sector [22]. This informal sector includes traditional healers (Kabiraj, Pir/Fakirs, and Hakims), homoeopathic practitioners, village doctors (Palli Chikitsoks), and pharmacies that provide both advice and allopathic medicine without requiring a prescription [24]. Village doctors were introduced into the Bangladeshi health scene in the late 1970s [25,26,27,28,29,30]. Modelled on the Chinese barefoot doctors, these village doctors were expected to provide a “new cadre of field level health personnel” [31] who had received limited training [22]. The training programme ended within a relatively short period of time and today many village doctors are “unqualified” in the sense that they have had no training while others have had some training in the form of courses run by pharmaceutical companies [21,32].

The informal sector is both marketed and unregulated, yet provides health services to a vast array of Bangladeshi. Further blurring the distinction between public and private provision of health is evident in the considerable NGO investment in the provision of health-related services. For example, BRAC (previously known as the Bangladesh Rural Advancement Committee) addresses poverty through providing health and other services. “It has a presence in most villages in the country, employs more than 120,000 people, and has trained 105,000 community health workers” [27]. This NGO involvement has led to increased awareness and knowledge of health and treatment options, particularly around diarrhoeal disease. Bangladesh is something of an exemplar here, with a very large indigenous NGO sector [33].

### 2.2. Changes in the Use of ICT to Access These Providers

There have been a number of studies of household health information seeking behaviour in Bangladesh, but they have not taken into account the growing importance of ICTs [34,35,36], Ahmed *et al.* [37] acknowledge the role of radio and television as sources of information but they do not report on the use of mobile telephones or the Internet. The 2007 and 2011 Demographic and Health Surveys in Bangladesh show that mobile phone ownership by rural households increased from 25% to 75% in just 5 years. Our survey results showed that in 2013, 88% of households had a phone owned by a resident within the household. While 72% of men surveyed owned phones, only 47% of women surveyed were in possession of a phone. Despite the high presence of mobile phones, the survey reveals the extent of poverty in Mizapur, Chakuria and Korail slum. Less than 20% of people surveyed were professionals or operated businesses. The majority of people surveyed were factory workers and semi-skilled labor, with the remaining operating in manual and unskilled work. Forty-seven percent of all people surveyed had not entered secondary school.

Bangladesh’s National Policy on ICTs has sought to develop a country-wide ICT infrastructure for mobile phones. In June 2013, there were 105 million active mobile phone subscribers. By September 2013, the Bangladesh Telecommunication estimated that this had increased to over 110 million mobile phone subscribers. Already in 2011, it was reported that 98% of all Bangladeshi adults had mobile phone coverage [38]. Bangladesh has done much to encourage the use of mobile phones including: its claim to have the cheapest mobile phone call rates in the world and its insistence, in 2012, that all basic handsets subsequently imported have a bangla keypad, with the intention that touchscreen and smartphones will also subsequently conform and, in 2012, the availability of 3G technology in mobile phones, which improves speed, efficiency and connectivity of mobile phones and enables uninterrupted video streaming [39]. As part of its vision for a “Digital Bangladesh”, in 2010, the Bangladeshi government commissioned the Union Information and Services Centers (UISCs) in all Union Parishads (or district councils). These “one stop e-service outlet(s)” aim to provide government and private services to all citizens [40].

The use of ICTs and mobile phones to enhance and facilitate the marketization of Bangladesh’s health system is also evident. There are six mobile phone providers in Bangladesh-Grameen Phone, Bangalink, Robi, Citycell, Teletalk, and Airtel–all of which compete through a series of special offers and customised services for customers. At the end of 2013, Grameen Phone accounted for approximately 50% of all subscribers, with Robi and Bangalink each accounting for about 25% of subscribers. The ubiquity of mobile phones and the multitude of uses was clear during our field research. As we were informed: “a rickshaw puller has a mobile phone, a van driver also has a mobile phone.” All six phone companies offer health help lines to their subscribers. Grameen Phone has a HealthLine Service Initiative. This is advertised on their website as a “24 h Medical Call Center manned by Licensed Physicians and accessible to all Grameenphone subscribers”. This round-the-clock service connects a caller to a licensed general physician. The service provides information on medical facilities and drugs, as well as the interpretation of laboratory results, medical consultations and advice during emergencies. Robi offers health advice connecting callers to a call center agent. Robi’s website promises: “The agents will be specialized doctors who will provide instant solution/suggestion to the queries or will call back to the customer if the solution is complex and needed to be consulted before answering”. Bangalink similarly allows subscribers to receive health counselling through a call center. The caller gets to speak to an agent, all of whom are “MBBS (Batchelor of Medicine and Batchelor of Surgery) professionals and specially trained to provide health issue related consultancy over phone”. Airtel similarly provides a round-the-clock Tele Health Service. It advises its subscribers that they can receive health information and advice from “Japan Bangladesh Friendship Medical Services”. It also offers a service–the Care and Health Menu-on health advice and beauty guidance. This can either be through SMS “Click and Read” or through video clips “See it, Hear it”. The costs of these help lines vary from Taka 33/10 s to Taka 96/10 s. How do all these services fit into the emerging health knowledge economy? To what extent do people actually use ICTs in relation to health information seeking and how is this changing the health knowledge economy?

### 2.3. Changes in the Health Knowledge Economy and Information Seeking

Understanding the evolving health knowledge economy and its impact on poor people relates to broader interests by researchers and planners on how new technologies might benefit poor households by expanding their options in critical areas that affect their wellbeing. Our overall approach to treating health information seeking as part of a health knowledge economy builds on conceptual frameworks first put forward by Wilson [41] to study how households seek information [42,43,44,45], their criteria for choice of information sources [46] and the role of mediators and technology [47,48]. A number of studies have identified the information priorities of the poor in a changing ICT landscape [49,50,51,52,53] and have found, in several countries, that poor households include health-related information as a high priority [54,55].

The literature specifically on health information behavior and ICTs has focused primarily on high income countries and on individuals. This work has pointed to the huge differences in exposure and access to e-health both within and between countries [56] in part because of the need for particular skills, resources and technology literacy to undertake health information seeking [57] and, in part, because of economic and cultural factors which influence health information seeking online [58]. The literature also points to the very wide variety of factors that influence search behaviors [57], including that health-seeking may not always improve health; that it is frequently part of a process rather than an activity undertaken for its own sake and that health information seeking is in turn shaping and changing patient-provider interactions.

Recent research on the changing knowledge economy has emphasized the need to understand how people assess the trustworthiness of sources of information. For example, a study by Hertzum *et al.* [59] shows that people need ways that enable them to assess source trustworthiness-access alone is not enough. Heath and Motta [60] also emphasize the importance people place on the degree to which they can trust an information source. This is highly relevant for health-related issues, where it has long been recognized that problems of information asymmetry-where the provider has an inherent knowledge advantage over the user–make issues of trust particularly important.

Almost no attention has been paid to the regulatory environment within which the changing health knowledge economy is operating. A key assumption is that regulation and associated institutional arrangements are lagging behind the rapid growth in the use of new sources of health information. For example, mobile phone companies and a range of market-based health service providers are increasingly developing new models for providing access to expert medical knowledge through the internet and/or mobile phones [61]. As yet, however, there is no clear regulatory regime which addresses issues of quality and competence in relation to e- and mHealth interventions. Similarly, the widespread availability of unmediated health-related messages through mass media, advertising and unregulated providers raises important questions about trust, accountability and regulation [62,63,64].

Given the changes in the health knowledge economy, the dynamic ways in which individuals and households undertake health information seeking and the rapid, unregulated injection of ICTs into complex health systems, actor-centric approach is useful for embracing and representing such a complex, multifaceted and emerging reality.

## 3. Developing an Actor-Centric Approach

An actor-centric approach can help map the flow of information and knowledge between the complex array of actors that make up the health knowledge economy. It accommodates the changes observed above, and represents potential vulnerabilities of poor people if providers of health information and advice are strongly influenced by interest groups, such as pharmaceutical companies. It indicates significant key roles undertaken by stakeholders in the regulatory environment in both government and the private sector and informs debate about the creation of an enabling environment for innovation which is also a safe environment that protects the public.

**Figure 1:**
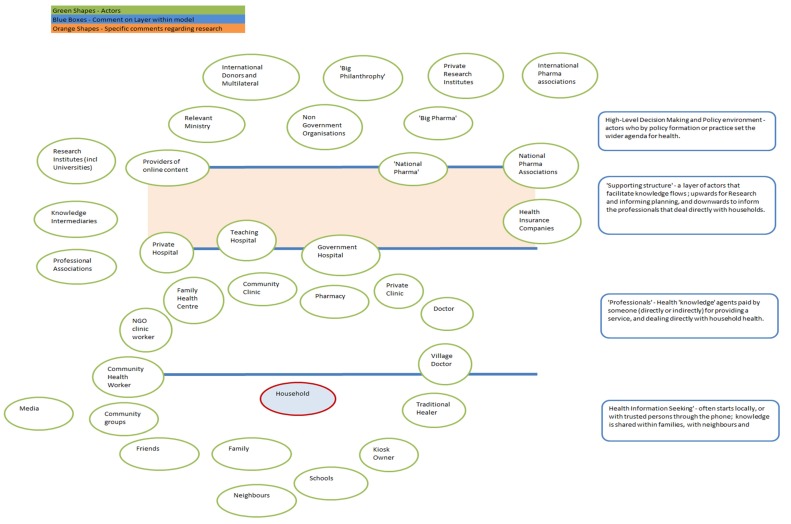
The different types of actors represented in the Bangladesh health system.

A first step in using an actor centric approach is to identify the generic types of actors in a typical health system. Situating this in Bangladesh, the different types of actors are presented in Figure 1. This has been developed through insights from the literature review, field research and stakeholder interviews to offer a composite view of potential sources of health information available to household members either for general health education or for health information which relates to specific symptoms.

Figure 1 does not seek to define distance nor information flow *per se*, but uses the relative position of the actors to each other, to represent possible closeness and influence. In a given situation, any actor may relate or communicate with any other actor. The physical representation of distance is indicative only. 

In mapping the relevant actors in the Bangladeshi health system, four layers of actors can be identified:

- High-level decision-making and policy environment (including government and political private sector actors)
- Supporting Structure of actors that facilitate knowledge flows
- Professional health knowledge agents
- Person-to-person engagement and trusted support.

## 4. Mapping a Bangladesh Case Study

**Figure 2:**
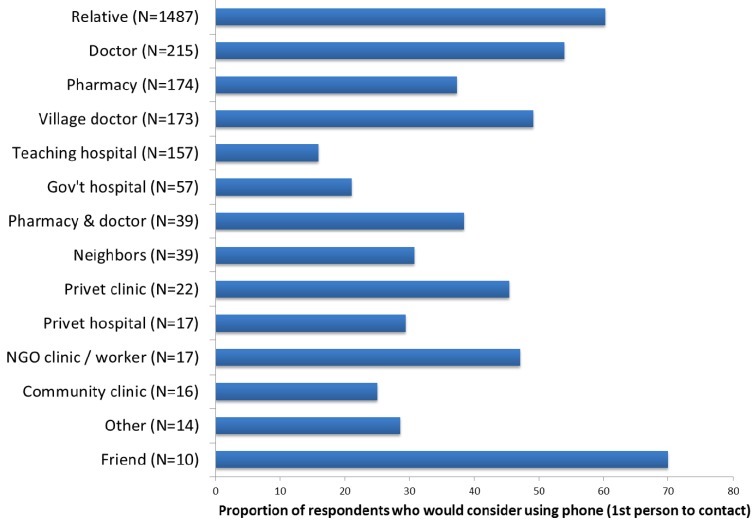
Proportion of respondents who would consider using their phone to make first contact when seeking health information. Authors survey 2014.

Our survey results showed that mobile phones can play a significant role in relation to navigating the Bangladeshi health system. In response to a hypothetical scenario, 50% of survey respondents indicated that they would consider contacting someone by phone and a further 1% said they would consider asking someone to call on their behalf. What is overwhelming clear in Figure 2 below, is that the majority of respondents would consider initiating their health information seeking process using their phones and contacting a relative.

In practice, however, when asked about their health information seeking behavior, only 26% of all respondents had used a phone for a health information-related task. These tasks included calling a doctor, village doctor, health worker or other health professional, checking on a doctor’s presence and availability, securing an appointment and receiving advice or making a complaint. Even fewer, only 23 people or 1% of survey respondents had actually used the health help lines described above.

The story of “Uddin’s wife” provided below was narrated by Uddin, head of household, in December 2013. It shows how, in practice, people use their phones to contact family members and outlines his navigation of a complex health system in rural villages and the city of Dhaka, rather than directly seeking phoning a health help line for information. The story started about 3 years ago–when mobile helplines were in their infancy.

In 2006, Uddin’s wife experienced problems during childbirth and their baby died during her delivery in their home village, Ratanpur.

**Figure 3:**
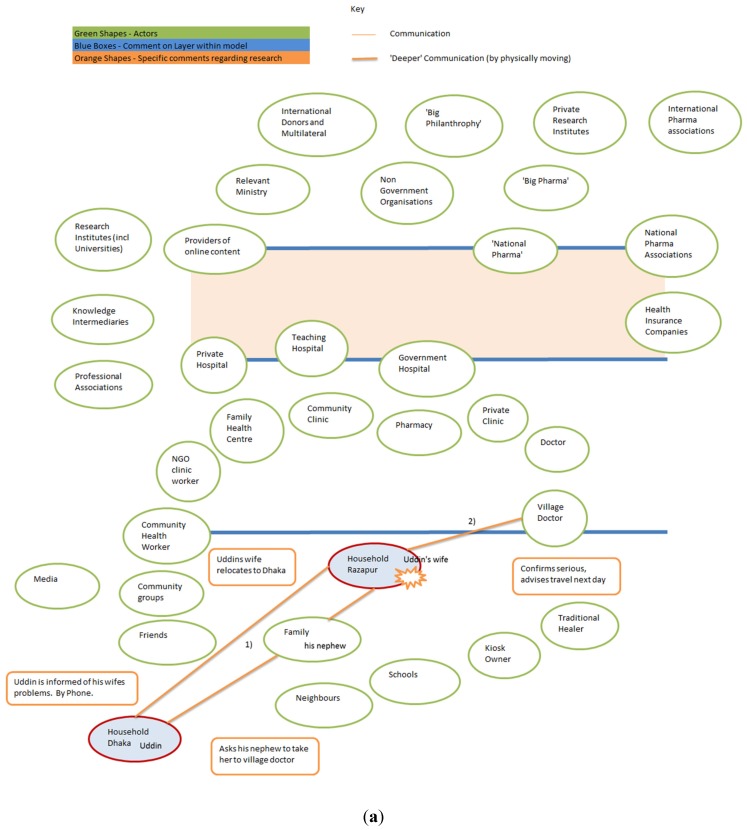

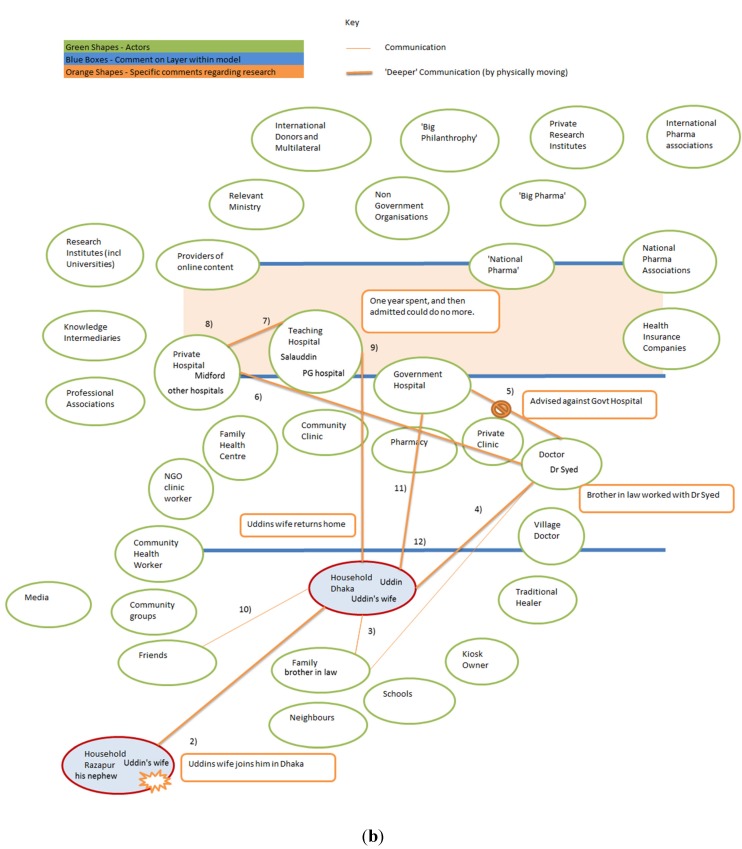
(**a**) Uddin’s story-Part 1 Identifying the health problem; (**b**) Uddin’s story-Part 2, the long road to hospital.

(1) Uddin was not at home and he asked his nephew to bring her to Dhaka to ensure that she received treatment.
(2) They took her to the village doctor, an informal health provider in the village, for treatment and reported to Uddin that her condition was serious and that they would only travel the following morning (see Figure 3a).
(3) When she arrived in Dhaka (continued in Figure 3b), Uddin consulted with his brother-in-law and
(4) Decided she should visit Dr. Syed in Jatrabari, Dhaka. He chose Dr. Syed because his brother-in-law worked with the doctor. Uddin’s brother in law said: “Dr. Syed will give what will be best”. Dr. Syed informed Uddin that she needed to go to hospital immediately. They discussed whether a government upazila Health Complex or private hospital would be best and
(5) Dr. Syed advised against an upazila health complex because of the long wait for an appointment.
(6) Dr. Syed provided Uddin with the address of a tertiary level government hospital, the Mitford in Dhaka, which offers a free service for the poor, where Uddin’s wife was admitted and had an MRI (Magnetic Resonance Imaging) scan, but where she received no treatment, so
(7) She went to Salauddin private hospital and
(8) Then spent a year at PG Government Tertiary Teaching hospital receiving treatment, until they admitted that they could do no more for her, but Uddin continued to seek treatment for his wife.
(10) While Uddin was in Malaysia, he discussed his wife’s illness with one of his fellow migrants. This migrant told Uddin about “his uncle who is a doctor who lives in Mohakhali” and who worked at the Government specialized cancer hospital. He then talked to his uncle about Uddin’s wife.
(11) Uddin sent a large sum of money to this doctor as an upfront payment, and this ensured treatment and medicine.
(12) After being released from this hospital, Uddin’s wife was better able to feed herself and walk around, but then she developed an ulcer on her leg and subsequent complications. She died before she was able to seek further treatment.

This narrative describes both information seeking and health care. While we are concerned mainly with health information seeking, as a case study it illustrates the many actors experienced as a part of health information and care seeking, and the role of the telephone.

Initially the story starts with the household in the village communicating with Uddin who is a migrant worker in Dhaka. In Figure 3 above, communication is represented by lines, where thicker lines represent face-to face-communication.

Figure 3a illustrates a standard use of the mobile phone, to call a relative for help. In this case, face-to-face assessment remains key, and so the unnamed wife and nephew approach the village doctor, and are given interim treatment to assist travel. The narrative then moves to Dhaka where a number of health providers are engaged.

As illustrated in Figure 3b, in Dhaka the story unfolds as a plethora of health providers and people are consulted for information and treatment. Once again the family is used to identify a relevant source and gatekeeper to a formal health provider in the form of Dr. Syed. He advised against using the upazila government hospital because of the long queues for appointment and instead suggested private and tertiary teaching hospitals for diagnostic facilities and treatment. The story continued with further advice coming from an acquaintance, and introductions being made to a specialized government hospital, with associated financial flows being an important key to opening the gates. This single case study illustrates the complexity of navigating Bangladesh’s health system. It shows that the mobile phone played an important role in terms of making appointments and seeking support, but does not include specific health helplines.

ICT plays a small but significant role in the story. A phone call from Uddin’s wife to her husband starts the process, and Uddin calls his nephew from Dhaka. Other phone calls are made to set up appointments and to confirm that finance has arrived and that travel is worth undertaking. In this story we do not find the participants referring to Telecom Helplines, however that is not to say that this would be true for a broader sample, nor that it might not become more embedded in the future.

## 5. What is the Role of ICTs in Health Knowledge Systems?

Given Uddin’s account of health information and health seeking, how might current mHealth activities be integrated within it? Yonazi *et al*. [65] suggest five opportunities for ICTs to support the health system, relating to the lack of skilled healthcare staff; health information system inadequacies; insufficient equipment, medication and other supplies; lack of information concerning preventable disease; and financial limitations. The following discussion examines each of the opportunities identified by Yonazi *et al*. [65] in turn, and each is followed with an analysis of how these opportunities can be applied to the healthcare system in Bangladesh.

- The lack of skilled healthcare staff: Yonazi *et al*. [65] suggest that “eLearning and telemedicine solutions can extend expertise to remote areas and provide otherwise inaccessible care”. This is a key role for the actor centric approach proposed in this paper, and which draws attention to asking who the healthcare workers are, and emphasizes both formal and informal providers. The Bangladesh heath system has community volunteers funded by NGOs, as well as long-standing village doctors. Pharmacists often offer advice. While it is accepted that ICTs could be used to strengthen the skill of healthcare workers, the identification of relevant healthcare workers who require this additional learning is a priority. In addition, as Malone *et al*. have shown, even in high-income contexts, the quality of access to health care can have a significant impact on online health information seeking with “ease of access” positively influencing information seeking [58]. In contexts such as Bangladesh where access to skilled health care is extremely difficult, the data suggests that online searching is currently not highly valued.
- Health information system inadequacies: “Data collection and surveillance mHealth applications can monitor and track health indicators in real time, providing insight to policymakers on true challenges and providing valuable data enabling health workers to better serve and patients to be more proactive in their own health” [65]. The feedback of data from the field to policy actors has long been recognized as important. But applying an actor centric model forces one to ask: which actors need to supply the data? Will only the formal system provide data, or would it be possible to engage with the informal healthcare workers to provide feedback that might make policy development more evidenced based and potentially more responsive to the needs of the poor? [56]. Village doctors are often, as shown in the above account, a critical entry point into the health system. Experimentation with using mHealth with village doctors in Bangladesh has not, to date, been successful and more work needs to be done for mHealth to offer a useful service to these informal health suppliers.
- Insufficient equipment, medication and other supplies: “Supply Chain Management mHealth applications can decrease stock-out frequency and increase efficacy of and trust in health system” [65]. An actor centric perspective focuses on who is providing drugs and equipment, rather than assuming that these are available only from formal providers. In Bangladesh, the challenge is not just the shortage of drugs and equipment but the attachment of good advice to the provision of drugs and equipment. Data from many emerging economies suggests that fake drugs can be killers, and, in Bangladesh, even a genuine drug taken for an inappropriate illness or in inappropriate dosage is a waste of limited resources and can do more harm than good [37]. In addition, although not explicitly developed in the above account of Uddin’s wife, it is evident that there is substantial scope for patients to receive different and multiple drugs from different providers and to not complete a course of medication.
- Lack of information concerning preventable diseases: “Public health promotion applications can be used to disseminate empowering information in a friendly, personal manner. Engaging without being intrusive” [65]. Our example shows the connectedness of society, and helps visualize the processes of health information seeking through the mapping of information flows. It is not enough to provide information on a Short Message System (SMS) text if no one reads the text. In many resource poor settings, a lack of literacy prevents the reading of a text by a significant portion of the population, and overuse of unsolicited texts has meant that many of those who could read the text no longer bother to. In addition, even though Uddin would have had several possible ways of accessing information through mHealth (phoning a health help line, reading government-sent SMS messages), at no point did he avail himself of these possibilities. An actor centric perspective encourages researchers to explore who talks to who, how they communicate and what kinds of information they are seeking.
- Financial limitations: “Health financing and personal insurance programmes offer increased opportunities for savings, both for patients and healthcare providers” [65]. ICT has opened up financial systems to resource poor communities. Uddin’s story illustrates how less-formalized financial flows compromise an integral part of the health information seeking, and ICTs can facilitate enhanced communication between people, thus aiding individuals in their search for health financing mechanisms, and enable more innovative financial flows between actors.

This list of five basic opportunities for eHealth to positively influence and support the health system is echoed by others. Hellström, [66], for example, supports similar opportunities for mHealth, namely: Education and awareness; Data collection and health record access; Disease/epidemic outbreak tracking; Health/administrative systems; Analysis, Diagnosis and consultation. From his list we once again see the importance of identifying who is the provider of information, and how much influence will they have on the health information seeking behavior of the individual and household. Uddin is not unique in this regard. Survey results show that 60% respondents start their exploration of the health system by contacting a relative. If the respondent has some seniority and authority within the household, then any choice of a relative as a first point of contact is driven by gaining information and compassionate support. Where the respondent is not the head of the household, the first choice of consultation with a relative is driven by the desire to gain “permission” to consult further, including accessing resources needed to consult within the health system. Whereas Yonazi *et al*. [65] see, as one of their five opportunities, eHealth as contributing to financial constraints and savings within the health system, Hellström identified monitoring and medication compliance as a possible eHealth activity. Mecheal [67] has also developed a similar list of components to which he adds treatment compliance as a basic opportunity for ICT. However as shown in our application of Bangladeshi actor-orientated data to these basic opportunities, this challenges us to examine the detail behind these broad categories of opportunity-who will send the compliance prompt, and how will it be received? Reception will be dependent on the trust and linkage the patient has with the specific health actor. Further challenges may include: how is information about disease prevention received and by whom? How does the supply of informal health provision interact with issues such as formal health system supply chain issues? Mecheal [67] also adds “emergency medical response” as a part of eHealth. The simple phone call summoning support can be vital. However whereas the assumption might be that such a call summons formal emergency services, an actor-centric understanding and the story of Uddin suggest that support from one’s own social network-before interacting with the formal health system-may be a critical addition.

While this section has not sought to undertake a comprehensive mHealth literature survey, it does demonstrate the significance of an actor-centric approach. Generic terms such as “public health promotion”, can be advanced through ICT, but also critically need to be contextualized. Who holds the trust or respect of the people enough that they might act upon given advice? What channels are seen and subsequently acted upon by the public? Where are people seeking their health information, both passively and actively? These are important questions to address.

## 6. Theory of Planned Behavior

As is evident from Figure 1 above, the landscape of actors provides numerous choices for households and individuals. However, the decision-making process for accepting or rejecting health information is in itself a distinct set of decisions that someone has to make either based on their own beliefs or on the value they put in others’ advice (see Figure 3). This means that not all avenues are always pursued. Advice or information may come from the many sources identified in our mapping of the health system, but the reliability of each source and the trustworthiness of their information will be assessed by the recipient.

The factors informing such a decisions can be identified using the theory of planned behavior. Azjben and Fishbein’s [68,69] widely-used theory of planned behavior relates individual beliefs to motivations, norms, intentions and outcome beliefs and behaviors. Each person holds outcome and control beliefs on which they may draw in order to create an intention to behave. This approach to planned behavior goes beyond individual theories of behavior change in several important respects. It treats individual belief and action as contextually and collectively embedded in household level processes of decision-making. This means that factors such as gender and age are critical in driving outcomes. As is evident in the account above, fewer women own mobile phones and Uddin both determines and manages his wife’s interactions with the health system. Additionally, in a context of rapid changes in technologies and the cost of access, people are altering their patterns of behavior. In general these behaviors are spreading from urban to rural areas and from the young to the old. “According to the theory of planned behavior, human action is guided by three kinds of considerations: beliefs about the likely outcomes of the behavior and the evaluations of these outcomes (behavioral beliefs), beliefs about the normative expectations of others and motivation to comply with these expectations (normative beliefs), and beliefs about the presence of factors that may facilitate or impede performance of the behavior and the perceived power of these factors (control beliefs)” [68,69].

There has been considerable work using this concept, and which we have drawn upon [70,71]. Ajzen, working with Madden and Ellen [72] found a considerable limitation in his original model called theory of reasoned action. In 1992 he reworked the model adding the control beliefs-those factors outside the control of the person and changed the name to theory of planned behavior (TPB). Since then studies on behavior change have used the theory extensively while noting its limitations.

Recent work has identified various factors influencing an individual’s decision-making and choices. These include past experience [73], avoiding past bad decisions [74], cognitive biases [75], age and individual differences [76], belief in personal relevance [77], and an escalation of commitment (all cited in [78]). All these are potentially captured in the theory of planned behavior outcome beliefs.

However the theory of planned behavior states that intention to behavior is also informed by reference to the social norm-and in a society such as Bangladesh the approach to health information seeking through a social lens is strong. We have seen that Uddin’s story starts with, and is peppered by, visits to family and friends and the seeking of advice and guidance from them. The value an individual puts on any particular social referent is likely to be high within Bangladesh society.

Using the theory of planned behavior in conjunction with an actor-centric approach enables a breakdown of the health knowledge economy, juxtaposing actor-orientated behavior across the different layers: high-level political and private sector economy; supporting structures; professional input into health seeking exchanges and person-to-person engagement and support. This, as demonstrated above, places emphasis on how people and households seek and identify useful health information; how decisions are made and how they navigate their way through complex arrangements and different levels of formal and informal health services.

## 7. Conclusions

Focusing on user demand and on how people find health information and make care-seeking decisions offers a different perspective on the versatility of e- and mHealth. To date, very little attention has focused on the user side of mHealth, on how, when and why people engage in online health information searching. The little that has been done in high-income countries [58,79] reveals the complexity and multi-faceted nature of information seeking and the difficulties of finding appropriate health information online [79]. Combining an actor-centric approach with the theory of planned behavior enables an exploration of the complexity of the health system, as well as an exploration of the intricacies of people’s health information seeking and decisions about treatment. Health provision in Bangladesh–and arguably in many other contexts–is undoubtedly not a linear flow from government to citizen. Rather, the health system in emerging economies, is multifaceted and convoluted; comprising of a mix of government and private provision, formal and informal providers, physical and informational provision, and informed and uninformed commentary [2]. Furthermore, a health knowledge economy and corresponding power differentials determine who has access to different forms of information. As the discussion above shows, informal relationships are extensively drawn upon to help facilitate access to health information and care seeking.

Although the landscape of actors provides numerous choices for households, the decision-making process for accepting or rejecting health information is a process of incremental decisions stemming from a combination of personal circumstances and engagement with the health system. The factors informing such decision-making can be identified using the theory of planned behavior. Each person holds outcome and control beliefs on which they may draw in order to create an intention to behave. However the theory of planned behavior states that intention to behavior is also informed by reference to the social norm–and the norm in Bangladesh is to seek information through relationships, networks and contacts. By combining the theory of planned behavior with an actor-centric approach, greater understanding of the health information seeking behavior of a society such as Bangladesh is achieved. This offers new ways to conceptualize the complexity of the health knowledge economy, through interactions within and between layers of policy, professional institutions, professionals and personal relationships. It also enables one to ask critical questions such as: Who holds the trust or respect of the people enough that they might take up any advice given? What channels of information are seen and used by the public? Where are people seeking their health information, both passively and actively? Knowing these things will, in turn, illuminate better ways in which ICTs might be used to support the health system and to developing new applications, which seek specifically to meet the needs of the poor and disenfranchised.
